# The Role of FRMD7 in Idiopathic Infantile Nystagmus

**DOI:** 10.1155/2012/460956

**Published:** 2011-08-29

**Authors:** Rachel J. Watkins, Mervyn G. Thomas, Chris J. Talbot, Irene Gottlob, Sue Shackleton

**Affiliations:** ^1^Department of Biochemistry, Henry Wellcome Building, University of Leicester, Leicester LE1 9HN, UK; ^2^Ophthalmology Group, School of Medicine, University of Leicester, P.O. Box 65, Leicester LE2 7LX, UK; ^3^Department of Genetics, University of Leicester, University Road, Leicester LE1 7RH, UK

## Abstract

Idiopathic infantile nystagmus (IIN) is an inherited disorder in which the nystagmus arises independently of any other symptoms, leading to the speculation that the disorder represents a primary defect in the area of the brain responsible for ocular motor control. The inheritance patterns are heterogeneous, however the most common form is X-linked. *FRMD7* resides at Xq26-27 and approximately 50% of X-linked IIN families map to this region. Currently 45 mutations within *FRMD7* have been associated with IIN, confirming the importance of *FRMD7* in the pathogenesis of the disease. Although mutations in *FRMD7* are known to cause IIN, very little is known about the function of the protein. FRMD7 contains a conserved N-terminal FERM domain suggesting that it may provide a link between the plasma membrane and actin cytoskeleton. Limited studies together with the knowledge of the function of other FERM domain containing proteins, suggest that FRMD7 may play a role in membrane extension during neuronal development through remodeling of the actin cytoskeleton.

## 1. Idiopathic Infantile Nystagmus

Nystagmus consists of rhythmic involuntary oscillations of the eyes and can occur in early childhood (infantile nystagmus) or can be acquired later in life (acquired nystagmus) [1, 2]. Idiopathic infantile nystagmus (IIN) has been found to be the most common type of nystagmus and has an estimated prevalence of 1.9 per 10,000 in Leicestershire and Rutland, UK [1]. Unlike other forms of nystagmus, IIN arises independently of any other visual or neurological abnormality. This has led to the speculation that the disorder represents a primary defect in regions of the brain responsible for ocular motor control [3, 4]. The impact of nystagmus on vision varies but can be significant due to the constant eye movement. Indeed visual function in many patients scores worse than in those with age-related macular degeneration [5].

## 2. Genetics of IIN

The inheritance patterns of IIN are heterogeneous and have been described as autosomal dominant (OMIM 164100) [6–8], autosomal recessive (OMIM 257400) [9] and X-linked (OMIM 31700). However, the most common form of inheritance is X-linked [10], which can be either dominant or recessive and X-linked loci have been identified at Xp11.4-p11.3 [11], Xp22 [12], and Xq26-q27 [10]. The gene responsible for IIN at the Xp11.4-p11.3 locus has not yet been identified. *GPR143* resides at Xp22 and mutations within it are primarily linked to ocular albinism (OA), where nystagmus results as a secondary phenotype [12]. However, GPR143 mutations have also been found to cause a variant form of OA with IIN as the most prominent and only consistent finding [13] and has been reported in IIN families, without the classical phenotype of ocular albinism [14]. This raises the possibility that GPR143 mutations may be more directly involved in the pathogenesis of IIN than first thought. However, albinism must be excluded as a factor by extensive clinical examination before it can be confirmed that Xp22 is an IIN locus. In approximately 50% of families, X-linked IIN maps to Xq26-q27 and has been shown to be linked to mutations in the *FRMD7* gene [4, 15, 16]. X-linked nystagmus pedigrees linked to the FRMD7 locus have shown either dominant or recessive inheritance patterns with a variable degree of penetrance in females [16, 17]. Possible mechanisms for the variability in penetrance include skewed X-inactivation, genetic modifiers (such as polymorphisms in interacting proteins), and other nongenetic influences on oculomotor development. These factors may also explain why both dominant and recessive X-linked pedigrees can show linkage to the same region [16].

Whilst skewed X-inactivation is a possible mechanism for the variable penetrance seen amongst females, the evidence for it remains controversial. Kaplan et al. demonstrated an increased susceptibility to skewed X-inactivation in clinically affected females harboring FRMD7 mutations when compared to their unaffected spouses [17], whilst Self et al. did not find a clear-cut difference in the pattern of X-inactivation between affected and unaffected carriers of the FRMD7 mutations [16]. In agreement with Kaplan et al., the vast majority of genes on the long arm of the X chromosome are subject to X-inactivation, including those immediately flanking *FRMD7* (MST4, MBNL3, and RAP2C) [16]. Since genes that are subjected to X-inactivation tend to be clustered into domains, it is very likely that *FRMD7* is also inactivated. If complete skewing is restricted to the specific cell lineages such as parts of the developing brain and retina (where most FRMD7 expression occurs), it is possible that complete skewing may be missed using DNA extracted from blood or saliva [16]. Currently, the basis of incomplete penetrance of the disease in females is not explained.

## 3. FRMD7 (FERM Domain-Containing 7) Domain Structure

**Figure 1:**
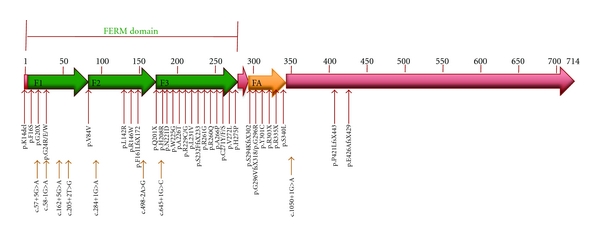
A schematic representation of the structure of the FRMD7 protein. It contains an N-terminal FERM domain (green) and FERM-adjacent (FA) domain (yellow). The FA domain consists of the 3 lobes F1 (lobe A), F2 (lobe B) and F3 (lobe C). The positions of IIN mutations within FRMD7 have been indicated. Many of the mutations cluster around the F3 lobe of the FERM domain and the FA domain.

The human *FRMD7* gene (ENSG000001656940) comprises 12 exons (ENST00000298542) and encodes a 714-residue polypeptide (ENSP00000298542). FRMD7 contains a conserved N-terminal FERM domain and FERM-adjacent domain, whereas the C-terminal region bears no significant homology to other proteins (Figure 1). FERM domains are characteristic of the band 4.1 superfamily and take their name from the 4.1 (four point one) and ERM (ezrin, radixin, and moesin) proteins in which they were first discovered [18]. The FERM domain of FRMD7 is located between amino acids 2–282 (ensemble, ENSP00000298542), whilst the FA domain is located between amino acids 288–336 (ensemble, ENSP00000298542) (Figure 1). FERM domains have 3 lobed “cloverleaf” structures, each lobe representing a compactly folded structure. Lobe A (also known as F1, the most N-terminal) has a fold resembling ubiquitin; lobe B (also known as F2, the central lobe) resembles acyl-CoA-binding proteins and lobe C (also known as F3, the most C-terminal) has a fold related to a pleckstrin homology domain/phosphotyrosine binding domain. The close packing of these domains suggests they do not function independently, but rather form a coordinated structure [19]. Ezrin, radixin, and moesin are a family of proteins that provide a link between the plasma membrane and the cortical actin cytoskeleton [20]. This suggests that FRMD7 may be involved in signal transduction between the plasma membrane and cytoskeleton. The FA region is found next to FERM domains in a subset of FERM containing proteins. This region is thought to be a regulatory adaption in these proteins, as it contains conserved motifs that are potential substrates for kinases and is the known regulatory phosphorylation site of 4.1 [19]. FARP1 and FARP2 are the closest homologues of FRMD7 [4]. As both of these proteins play roles during neuronal development [21–23], it has been suggested that FRMD7 may also regulate this process.

## 4. Mutation of FRMD7 Is Associated with IIN

To date, 45 different mutations within FRMD7 have been reported in IIN patients (Table 1), 79% of which are unique and have only been identified in one IIN family. The mutations c.284+1G>A, c.425T>G (p.L142R), and c.1003C>T (p.R335X) are the most common and have each been detected in 3 different families. The mutations concentrate heavily within the FERM and FA domains, suggesting that these domains play important roles in the function of FRMD7.

Just under half (42%) of the mutations identified within *FRMD7* are predicted to cause gross defects at the protein level due to introduction of frameshift, nonsense mutations, and/or aberrant splicing. The latter may also lead to nonsense-mediated decay of the mRNA. When the nonconserved splice site mutation c.162+5G>A was investigated, negligible amounts of transcript were detected compared with controls [4]. In contrast, the apparently silent variant, G252A (V84V), creates a new splice acceptor site within exon 4 that in lymphocytes results in the loss of transcript containing the sequence of exons 1–5 and the rare presence of a transcript with skipping of exon 4 [4].

The other half (53%) of the mutations identified within *FRMD7* are missense. These mutations may disrupt FRMD7 function by destabilizing the protein, disrupting binding sites with interacting partners and/or preventing regulatory modifications to the protein such as phosphorylation and/or glycosylation. When the effects of the mutations p.G24R, p.L142R, p.A266P, and p.C271Y were modelled by Tarpey et al. on the three-dimensional structure of the cytoskeletal protein 4.1R (the closest homologue of FRMD7 with known structure), they found that the missense mutations p.G24R, p.L142R, and p.C271Y are likely to destabilize the protein by the introduction of larger amino acids within restricted areas of the protein. It was also predicted that the introduction of a proline residue at position 266 (p.A266P) might disrupt a helical region in the F3 lobe of the FERM domain [4]. Similarly, the histidine at codon 208 is one of two amino acids located inside the region between two *β*-sheets, and substituting it with an arginine is likely to destabilize the protein by introducing a larger amino acid within a restricted area of the protein [24]. However, these predictions have yet to be verified experimentally.

In addition to the mutations listed in Table 1, a large intragenic deletion of *FRMD7* that spans exons 2, 3, and 4 has been reported in a pedigree with 2 separate X-linked traits, idiopathic infantile nystagmus, and deuteranomaly. The *FRMD7* mutation is associated with the family's nystagmus. It is hypothesised that this deletion eliminates 227 nucleotides of the *FRMD7* gene causing a frame shift that alters 19 amino acids before premature termination at codon 39. Further work sequencing the *FRMD7* gene across the mutated area (including coding and noncoding regions) would need to be carried out in this family to confirm the exact location of the deletion.

## 5. FRMD7 mRNA Expression in the Developing Brain

Initially, Tarpey et al. employed conventional RT-PCR to examine the expression pattern of FRMD7 in human adult and fetal tissues (kidney, liver, pancreas, heart, and brain). They found that FRMD7 is expressed in all adult tissues examined; however, the levels of expression were lower in the heart and brain. In the human fetal tissues examined, FRMD7 mRNA was only detected in kidney [4]. This suggests that FRMD7 is only expressed in the fetal kidney. However, in the same study, a further investigation using in situ hybridization revealed that FRMD7 mRNA is expressed in human embryonic brain at days 37 and 56 postovulation [4]. In addition, a study by Self et al., which investigated the expression of FRMD7 mRNA in murine heart lung and brain during early development stages (ED11-PD8) using real-time PCR analysis, has shown that FRMD7 is expressed in these tissues [32]. It is likely that Tarpey et al. missed FRMD7 mRNA expression in human fetal heart and brain when they employed the less sensitive technique of RT-PCR. This hypothesis is supported by data from Self et al., which shows that in murine heart and lung (but not brain) FRMD7 mRNA expression is low (at limits of detection) during the early developmental stages (ED11-PD8) and increases significantly in adulthood [32].

Interestingly, Self et al. found that the expression profile of FRMD7 mRNA during early development (ED11-PD8) is different in murine brain samples compared to heart and lung. In the brain, FRMD7 mRNA expression was similarly low at early time points (ED11–17); however, there was a marked increase in the expression at ED18. A higher level of expression was maintained until adulthood (PD1, PD8, and adulthood) [32]. Embryonic mouse brain development starts approximately 10-11 days after gestation (ED10-ED11). By embryonic day 18 (ED18), neurons start to send out axons and dendrites poised for synaptic connections [33]. The approximate 5-fold increase of FRMD7 mRNA at ED18 suggests that it is involved in this process [32]. Further to this, when Matsuki et al. examined the expression profiles of 397 genes related to neuronal development in ED12, 15, 18, and postnatal day 0 mouse brains, they found that expression of 14.9% of the genes peaked at ED18. The genes that peaked at ED18 have functions related to survival and growth, synapse formation and function, and determination and differentiation [34]. As FRMD7 has a similar temporal expression to this set of genes, it suggests that FRMD7 may also play a role in these processes.

In addition to determining that FRMD7 mRNA is expressed in human embryonic brain at 37 and 56 days postovulation, Tarpey et al. employed in situ hybridization to reveal that its expression is restricted and localized to certain areas. At 56 days postovulation, FRMD7 expression was seen in the ventricular layer of the forebrain, midbrain, cerebellar primordium, spinal cord, and developing neural retina [4]. In contrast, at 37 days postovulation, expression was restricted to the mid- and hindbrain [4]. These regions are known to be involved in motor control of eye movements, suggesting that FRMD7 may be involved in this process. As FRMD7 shows high sequence homology to FARP2 [4], which is involved in neuronal development [21, 35], it has been speculated that FRMD7 may be involved in neuronal development in these regions of the brain [4].

Following on from this study, a more detailed examination of FRMD7 mRNA expression in the cerebral cortex during human embryo development (Carnegie Stage (CS)15, CS16, CS19, CS22, and CS23) and human fetal development (9 weeks postconception (wpc) and 14 wpc) by in situ hybridization was undertaken [3]. Strong hybridization signals were observed in the ventricular zone (VZ) of the forebrain at CS16 and CS19, in both the telencephalon and diencephalon. At CS22 and CS23, FRMD7 mRNA, remained expressed in the VZ, but it was also observed in the intermediate zone and cortical plate. By 9 and 14 wpc, limited cells in the ventricular layer showed expression of FRMD7 mRNA whilst the majority of FRMD7 expression was observed in the cortical plate and subplate [3]. During early development of the brain (prenatally) immature neurons generated from the final mitotic division of the neuronal cell progenitors begin to migrate from the VZ to the cortical plate using processes of radial glial cells as a guide [36]. These data suggest that FRMD7 may play a role in the radial migration of newborn neurons in the cerebral cortex during human embryo development. 

In a recent study, we identified expression of FRMD7 mRNA within specific neural substrates such as the developing afferent and efferent arms of vestibulo-ocular and optokinetic reflex. In addition, an interesting expression pattern was observed within the developing cerebellum (rhombomere 1) and the VZ of rhombomeres 2, 3, and 4 which would give rise to the vestibular nuclei (the horizontal neural integrator site) [37]. These expression patterns correlate well to the phenotype observed. For instance, we identified that *FRMD7* mutations are causative of familial periodic alternating nystagmus and in affected patients the optokinetic reflex was absent [37]. Similarly, in unaffected carriers with *FRMD7* mutations, a subnormal gain was observed for the optokinetic reflex [37].

## 6. FRMD7 Protein Expression in the Developing Brain

In keeping with the mRNA expression data, immunohistochemical analysis of the developing mouse cerebral cortex revealed that there was strong immunoreactivity of FRMD7 protein in the ventricular and intermediate zones at early stages of development (ED13 and ED15), whereas at later development stages (ED17 and P0), it appears to be restricted to the cortical plate [3]. High levels of FRMD7 protein have also been detected in the brainstem (pons, medulla, and oblongata) in the human fetal brain at 16-17 wpc, indicating that its expression is not restricted to the cortex [38]. This area of the brain is also an important region associated with ocular motor control.

When the expression of FRMD7 protein in early post-natal tissues was investigated by Betts-Henderson et al. they found that it is highly expressed in eye and brain (cortex, hippocampus, cerebellum, and olfactory bulb). In contrast, little or no expression was observed in the liver, kidney, skeletal muscle, and heart muscle [3]. It is, therefore, speculated that FRMD7 may also play a role in the adapting postnatal brain and eye. The brain tissues examined in this study consisted of many cell types including glial cells and neurons. FRMD7 is expressed in cultured cortical neurons and its expression level increases at later times in culture. The upregulation of FRMD7 at later passages suggests that it may play a role in the maturation and morphological differentiation of neurons [3]. In agreement with this hypothesis, FRMD7 mRNA levels are rapidly and significantly increased (*P* = 0.0008, 12 hours post differentiation) in differentiating Neuro2A cells when compared with undifferentiated cells [3]. Moreover, an increase in FRMD7 protein expression was also observed within 12 hours of differentiation [3].

## 7. FRMD7 Promotes Neurite Elongation and May Regulate Neuronal Actin Dynamics at the Growth Cone

FRMD7 protein is highly colocalised with actin within the cell body of both undifferentiated and differentiated Neuro2A cells. In addition, in differentiated Neuro2A cells, FRMD7 is observed in the neurite processes and growth cones. FRMD7 is highly localized to the actin-rich regions of the primary neurite extension but is almost absent from the actin-rich secondary extensions arising from the primary neurite. Within growth cones, FRMD7 is present at the actin-rich distal end [3]. The C-terminus of FRMD7 has been shown to play a key role in the subcellular localization of FRMD7 in Neuro2A cells as mutant proteins lacking the C-terminal domain or containing the c.1003C>T (p.R335X) mutation exhibited a primarily nuclear distribution [38]. 

Knockdown of FRMD7 protein expression in nondifferentiated Neuro2A cells results in an increase in the number of cells with neurites, in the average number of neurites per cell and in the percentage of cells with neurite branching. The same is true in differentiated Neuro2A cells; however, these cells also exhibit a large reduction in average neurite length. These data further suggest FRMD7 plays an important role during neuronal development, specifically elongation of axons and dendrites [3]. 

Neurite formation, branching and elongation all require spatial and dynamic reorganisation of the cytoskeleton. If FRMD7 regulates these processes, it must be able to coordinate remodeling of the actin cytoskeleton. Indeed the absence of FRMD7 in differentiated Neuro2A cells results in a noticeable increase in F-actin [3]. This increase in polymerized actin is reflected by an increased number of neuritis per cell as well as increased filopodia and lamellipodia in the neurite processes. 

FRMD7 protein is expressed at the actin-rich distal ends of growth cones, and it promotes the elongation of neuritis, suggesting that it may regulate growth cone guidance. One of the most crucial steps in the formation and movement of the neuronal growth cone is the recruitment and activation of the Rho family of small GTPases (Rac1, Cdc42, and RhoA) and their regulators, the Rho guanine nucleotide exchange factors (GEF) and GTPases activating proteins (GAP) [39]. This is because the Rho GTPases are key regulators of actin cytoskeleton dynamics [40]. It remains to be determined whether FRMD7, like FARP1 and FARP2, functions as a GEF [21–23]. However, the FERM domain-containing protein radixin is known to be an upstream regulator of Rho family members, interacting with both inhibitory regulators (e.g., Rho GDI) and stimulatory regulators (e.g., Dbl) [41]. Therefore, it is possible that FRMD7 is involved in the regulation of neuronal cytoskeletal dynamics through Rho GTPase signaling at the growth cone [3].

## 8. Summary

Currently, 45 mutations in FRMD7 have been associated with IIN almost all concentrate in the conserved FERM and FA domain. Although very little is known about FRMD7 function, expression analysis and known functions of related proteins suggest that it may carry out roles related to neuronal development. Currently, FRMD7 is thought to participate in pathways that control survival and growth, synapse formation and function, determination and differentiation (axogenesis and dendritogenesis), asymmetrical neuronal precursor cell division, and the radial migration of newborn neurons. More specifically, it is thought that FRMD7 is involved in the regulation of neuronal cytoskeletal dynamics at the growth cone through Rho GTPase signaling. Mutations in FRMD7 may prevent the recruitment and the activation of the Rho family of small GTPases (Rac1, Cdc42, and RhoA) and their regulators, the Rho GEFs and GAPs. In a similar fashion to knockdown of FRMD7 expression, mutation of FRMD7 in IIN may prevent elongation of neurite processes during differentiation. In addition, mutations in FRMD7 may prevent axons from changing direction in response to stimuli. Nystagmus may, therefore, result from defective axogenesis, dendritogenesis, and neuronal guidance in the areas of the brain which control eye movements.

## Figures and Tables

**Table 1:** A list of the FRMD7 mutations associated with idiopathic infantile nystagmus (IIN). Del: deletion, Ins: insertion, T: truncation, N: nonsense, M: missense, and S: splice.

Mutation		Class	Exon/intron affected	Reference
DNA	Protein			
c.41_43delAGA	p.K14del	del	Exon 1	[25]
				[4]
c.47T>C	p.F16S	M	Exon 1	[37]
c.57+5G>A		S	Intron 1	[15]
c.58-1G>A		S	Intron 1	[37]
c.58C>T	p.Q20X	N/T	Exon 2	[15]
c.70G>A	p.G24R	M	Exon 2	[25]
				[4]
c.70G>T	p.G24W	M	Exon 2	[26]
c.71G>A	p.G24E	M	Exon 2	[4]
c.162+5G>A		S	Intron 2	[4]
c.205+2T>G		S	Intron 3	[4]
c.252G>A	p.V84V	S	Exon 4	[4]
c.284+1G>A		S	Intron 4	[4]
				[16]
c.425T>G	p.L142R	M	Exon 6	[27]
				[4]
c.436C>T	p.R146W	M	Exon 6	[25]
c.479_480insT	p.F161LfsX172	Ins/T	Exon 6	[4]
c.498-2A>G		S	Intron 6	[15]
c.601C>T	p.Q201X	N/T	Exon 7	[4]
c.623A>G	p.H208R	M	Exon 7	[24]
c.645+1G>C		S	Intron 7	[4]
c.661A>G	p.N221D	M	Exon 8	[4]
c.673T>G	p.W225G	M	Exon 8	[15]
c.676G>A	p.A226T	M	Exon 8	[4]
c.685C>T	p.R229C	M	Exon 8	[25]
c.685C>G	p.R229G	M	Exon 8	[17]
c.691T>G	p.L231V	M	Exon 8	[4]
c.694_695delAG	p.S232FfsX233	del/T	Exon 8	[26]
c.781C>G	p.R261G	M	Exon 9	[28]
c.782G>A	p.R260Q	M	Exon 9	[26]
c.796G>C	p.A266P	M	Exon 9	[4]
c.811T>A	p.C271S	M	Exon 9	[37]
c.812G>T	p.C271F	M	Exon 9	[26]
				[29]
c.812G>A	p.C271Y	M	Exon 9	[4]
c.814G>T	p.V272L	M	Exon 9	[30]
c.824A>C	p.H275P	M	Exon 9	[15]
c.880_881insA	p.S294KfsX302	Ins/T	Exon 9	[16]
c.886G>C	p.G296R	M	Exon 9	[28]
c.887delG	p.G296VfsX318	del/T	Exon 9	[4]
c.902A>G	p.Y301C	M	Exon 9	[4]
c.910C>T	p.R303X	N/T	Exon 10	[26]
c.1003C>T	p.R335X	N/T	Exon 11	[4]
				[28]
c.1019C>T	p.S340L	M	Exon 11	[4]
c.1050+1G>A		S	Intron 11	[4]
c.1262delC	p.P421LfsX443	del/T	Exon 12	[4]
c.1275_1276delTG	p.E426AfsX429	del/T	Exon 12	[31]
